# Effects of exercise, cognitive, and dual-task interventions on cognition in type 2 diabetes mellitus: A systematic review and meta-analysis

**DOI:** 10.1371/journal.pone.0232958

**Published:** 2020-05-14

**Authors:** Samuel Cooke, Kyla Pennington, Arwel Jones, Chris Bridle, Mark F. Smith, Ffion Curtis

**Affiliations:** 1 Lincoln International Institute for Rural Health, University of Lincoln, Lincoln, United Kingdom; 2 School of Psychology, University of Lincoln, Lincoln, United Kingdom; 3 School of Psychology, University of Bedfordshire, Luton, United Kingdom; 4 School of Sports and Exercise Science, University of Lincoln, Lincoln, United Kingdom; Nottingham Trent University, UNITED KINGDOM

## Abstract

**Introduction:**

Previous evidence has shown significant effects of exercise, cognitive and dual-task training for improving cognition in healthy cohorts. The effects of these types of interventions in type 2 diabetes mellitus is unclear. The aim of this research was to systematically review evidence, and estimate the effect, of exercise, cognitive, and dual-task interventions on cognition in type 2 diabetes mellitus.

**Method:**

Electronic databases including PubMed, EMBASE, CINAHL, PsycINFO, SPORTDiscus, and MEDLINE were searched for ongoing and completed interventional trials investigating the effect of either an exercise, cognitive or dual-task intervention on cognition in type 2 diabetes mellitus.

**Results:**

Nine trials met the inclusion criteria–one dual-task, two cognitive, and six exercise. Meta-analyses of exercise trials showed no significant effects of exercise on measures of executive function (Stroop task, SMD = -0.31, 95% CI -0.71–0.09, *P* = 0.13, trail making test part A SMD = 0.28, 95% CI -0.20–0.77 *P* = 0.25, trail making test part B SMD = -0.15, 95% CI -0.64–0.34 *P* = 0.54, digit symbol SMD = 0.09, 95% CI -0.39–0.57 *P* = 0.72), and memory (immediate memory SMD = 0.20, 95% CI -0.28–0.69, *P* = 0.41 and delayed memory SMD = -0.06, 95% CI -0.55–0.42, *P* = 0.80). A meta-analysis could not be conducted using cognitive or dual-task data, but individual trials did report a favourable effect of interventions on cognition. Risk of bias was considered moderate to high for the majority of included trials.

**Conclusions:**

Meta-analyses of exercise trials identified a small effect size (0.31), which whilst not significant warrants further investigation. Larger and more robust trials are needed that report evidence using appropriate reporting guidelines (e.g. CONSORT) to increase confidence in the validity of results.

**Trial registration:**

Protocol was registered (CRD42017058526) on the International Prospective Register of Systematic Reviews (http://www.crd.york.ac.uk/PROSPERO).

## Introduction

Diabetes mellitus is a group of metabolic disorders characterised by hyperglycemia and caused by defects in insulin production, insulin action or both [1]. The number of diabetes cases worldwide have rapidly increased over the last four decades, rising from 108 million in 1980 to 422 million in 2014 [2]. Diabetes is a leading cause of mortality [3] and is a strong risk factor for both microvascular and macrovascular complications with growing evidence suggesting an association with cognitive dysfunction [4–8].

Type 2 diabetes mellitus (T2DM) is associated with an increased risk of cognitive dysfunction. Deficits in several cognitive domains that are affected in mild cognitive impairment and dementia have been observed in those with T2DM [9–13]. For example, a previous meta-analysis that included a total of 26,137 participants across 24 trials [11] reported that, compared to those without diabetes, individuals with T2DM showed an overall worse performance in tasks of attention/concentration (*d = -*0.19), visual memory (*d* = -0.26), verbal memory (*d = -*0.28), processing speed (*d = -*0.33), executive function (*d =* -0.33), and motor function (*d = -*0.36). The exact underlying mechanisms precipitating cognitive dysfunction in T2DM remain unclear. Evidence has shown poor glyceamic control to be strongly associated with the development of cognitive dysfunction [14, 15]. Cerebral and peripheral vascular complications that develop as a consequence of chronic exposure to hyperglycemia (e.g. neuropathy, white matter disease, stroke, myocardial infarcts, peripheral artery disease) have been linked to cognitive impairment in T2DM [16, 17]. Other proposed mechanisms such as chronic low-grade inflammation, insulin dysregulation, and vascular dysfunction have also been implicated in the development of diabetes-associated cognitive dysfunction [18, 19].

Numerous strategies to prevent cognitive dysfunction have been explored in healthy cohorts. Amongst these, several meta-analytic studies have shown both exercise [20–22] and cognitive training [23–25] to provide important cognitive benefits. Exercise has been shown to have a positive effect on T2DM related outcomes, including improving glucose control, reducing inflammation, improving insulin sensitivity, and reducing cardiovascular risk [26], all of which are factors known to affect cognition [19, 27]. Other mechanisms through which exercise has been proposed to improve cognition involve anatomical and biochemical adaptations such as reduced cerebral atrophy, increased neurogenesis, improved insulin signalling, enhanced cerebral blood flow, and the increased availability of neurotrophins and neurotransmitters [28–32]. Similar physiological mechanisms are also evident as a result of cognitive training alongside neural mechanisms including improved resting state neural activity and enhanced functional connectivity in the default mode network and central executive network [33]. The engagement in simultaneous exercise and cognitive training (dual-task training) has been shown to improve cognition beyond the effects of the single underlying components [34–36], suggesting that the combined effects of these tasks may have a potential additive effect on brain and physiological function.

Whilst no previous review has evaluated the effects of cognitive or dual-task training in T2DM, researchers have reviewed the effects of exercise training in this patient population. Two previous reviews [37, 38] present findings that support the effects of exercise on cognition in T2DM, reporting improvements is several cognitive domains including executive function, memory, attention, language, visuospatial ability and global cognition. One review [37] concluded that the beneficial effects of exercise may be most significant in brain regions that are most vulnerable to the process of ageing, specifically regions associated with executive function such as the prefrontal and frontal lobe. Conversely, the two most recent reviews [39, 40] suggest that the strength of the current available evidence does not support these conclusions. A limitation of these previous reviews were that they included a broad range of study designs and not one statistically quantify the effects of interventions. The current review will be the first to synthesis data from interventional trials. Conducting a robust synthesis of available evidence will reduce uncertainty about the effects of exercise, cognitive and dual-task interventions on cognition in T2DM. This will inform future interventions with respect to non-pharmacological prevention strategies, targeting cognitive impairment in diabetic populations. The primary aim of this research was to systematically review the evidence, and estimate the effects, of exercise, cognitive, and dual task interventions on cognition in T2DM.

## Method

Methods of analysis and eligibility criteria were specified in advance and documented in a protocol (CRD42017058526) registered on PROSPERO (International Prospective Register of Systematic Reviews; www.crd.york.ac.uk/PROSPERO/). This systematic review and meta-analysis was conducted in accordance with the PRISMA check-list, see S1 File.

### Eligibility criteria

Trials were considered for inclusion in this review subject to the following criteria being met. **Participants:** adults aged 18+ diagnosed with T2DM. **Intervention:** Any structured exercise, cognitive, or dual-task intervention. The design of the trial must have been such that the independent effects of either exercise, cognitive or dual-task training on cognition could be analysed. Dual-task trials were eligible only if the intervention consisted of the simultaneous engagement of exercise and cognitive activities (e.g. treadmill walking whilst performing a memory task) and not the combination of the two single underlying components (e.g. treadmill walking followed by memory training). **Comparison:** Any concurrent control group was eligible, including no contact/usual care, waiting list, sham exercise, passive training, or alternative active treatment. **Outcome:** Any validated neuropsychological test of cognition reported at baseline and follow up after exposure to either an exercise, cognitive or dual-task intervention. **Study design:** Any trials that allocated individuals to either an intervention or concurrent control group.

### Search strategy

The following electronic databases were searched for completed trials: PubMed, EMBASE, CINAHL, PsycINFO, SPORTDiscus, MEDLINE, and Health Technology Assessment (HTA). ClinicalTrial.gov and Cochrane Register of Controlled Trials were searched for ongoing trials. Conference Papers Index was searched for conference papers and abstracts, and Cochrane, PROSPERO, and the Database of Abstracts of Reviews of Effects (DARE) were searched for completed or ongoing systematic reviews. Database searching was supplemented by contact with study authors and research groups, forward and backward citation tracking from included trials or previous relevant reviews, with further searching via Google Scholar. Searches were conducted from database inception to March 2020. No limits on language or publication status were set.

Key search terms for database searching included the following (“Type 2 diabetes mellitus” OR “Non-insulin dependent diabetes mellitus” OR “Adult-onset diabetes mellitus,”) AND (“Exercise” OR “Physical activity” OR “Cognitive training” OR “Brain training” OR “Dual-task” OR “Motor-cognitive”) AND (“Cognition” OR “Neurocognitive function” OR “Brain function)”. An example search strategy for PubMed is provided in S2 File. All key search terms were combined, where possible, with medical sub-headings (MeSH) and indexed terms to identify potentially relevant studies. Retrieved trials were collated and stored using Endnote referencing software (EndNote X8, Clarivate Analytics, Philadelphia, USA). Duplicate citations were removed prior to the independent screening of title and abstract in accordance with the pre-specified eligibility criteria (S.C). Full text articles were retrieved for all trials that were not excluded based on title and abstract before independently screened for final eligibility (S.C & F.C). All discrepancies were resolved through further discussion, or where required, a third reviewer (K.P).

### Data abstraction

Data were extracted using an adapted Cochrane Data Extraction Template for interventions. Trial characteristics were extracted from each included trial based upon 1) Trial characteristics (trial aim, trial design, inclusion/exclusion criteria, sample size and methods of allocation), 2) Participant characteristics (diabetes diagnosis, age, sex, body mass index, length of diabetes diagnosis, medication), 3) Intervention/comparison (type, duration, frequency, intensity, length, delivery of intervention/control, site of delivery), and 4) Outcome measurements (all relevant cognitive outcomes and measurement tools). S.C undertook data extraction for each trial, with cross checking taking place by F.C. All discrepancies between reviewers in certain trials were resolved through further discussion, or where required, a third reviewer (K.P).

### Risk of bias assessment

S.C and F.C independently assessed the risk of bias for included trials using the Cochrane Risk of Bias assessment tool with the following domains: random sequence generation, allocation concealment, incomplete outcome data, blinding of outcome assessment and selective outcome reporting. Each domain was categorised as either low, unclear, or high with the risk of bias for each trial classified using the following criteria 1) low risk of bias (all criteria graded as low), 2) moderate risk of bias (one criterion graded as high or two criteria graded as unclear), and 3) high risk of bias (more than one criterion graded as high, or more than two graded as unclear) [41]. Disagreements between reviewers were resolved through further discussion, or where required, a third reviewer (K.P).

### Data analysis

Trials were pooled based on the intervention type (e.g. exercise, cognitive, or dual-task) and a separate set of analyses performed to quantify their effect on cognition in T2DM. Cognitive outcomes were grouped based on the cognitive domain measured (e.g. global cognition, executive function, memory, attention) and meta-analyses conducted on subdomains using compatible neuropsychological tasks (e.g. the Victorian Stroop task and the Stroop task were used to measure inhibition response, a subdomain of executive function). Change from baseline values were used to conduct meta-analyses as it allowed the comparison of more trials. Where trial data were presented as pre and post, change from baseline scores were calculated by deducting the baseline score from the follow up score. Standard error (SE) scores were converted to standard deviation (SD) scores using the following equation [42].

SD=SEx√N

Change from baseline standard deviation (SD) was calculated using the following correlation coefficient equation [42].

$$SD_{E/change}=\surd \;SD^{2}{}_{E/baseline}+SD^{2}{}_{E/final}-\left(2x0.5xSD_{E/baseline\;x}SD_{E/final}\right)$$

All meta-analyses were performed using Review Manager Version 5.3. Data were quantified using standardised mean difference (SMD) and 95% confidence intervals for continuous outcomes. A higher score that reflected a greater task performance was represented by a positive effect estimate. A lesser score that reflected a greater task performance was represented by a negative effect estimate. A random-effects model was chosen due to the expected heterogeneity between trial protocols. Heterogeneity was measured using Higgins I^2^ statistic [43]. An I^2^ threshold of >40% was set to detect heterogeneity.

## Results

### Search results

**Fig 1:**
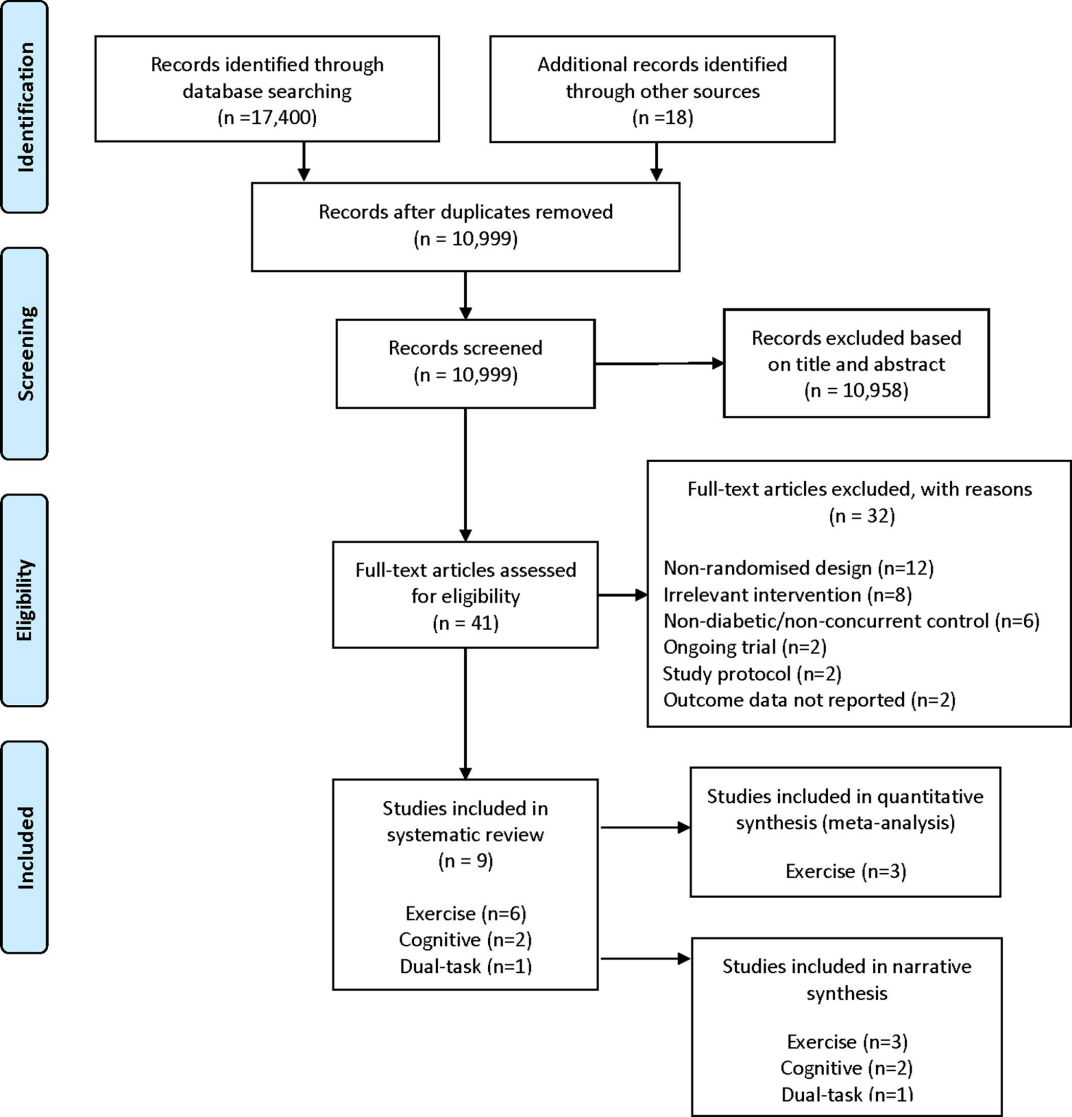
Study flow chart [53].

The search of all electronic databases provided a total of 17,400 distinct citations with an additional 18 citations identified through other sources (Fig 1). After adjusting for duplicated citations, 10,999 citations remained. Of these, 10,958 citations were discarded after reviewing for title and abstract as these records did not meet the pre-specified eligibility criteria. Of the remaining 41 citations, full texts were obtained and examined in detail for inclusion in this review. In total, 32 of these trials did not meet the pre-specified inclusion criteria for reason including non-randomised design (n = 12), irrelevant intervention (n = 8), non-diabetic/no concurrent control (n = 6), ongoing trial (n = 2), study protocol (n = 2), and outcome data not reported (n = 2), (Fig 1). Nine trials met the pre-defined inclusion criteria; six trials investigated the effect of an exercise intervention [44–49], two trials investigated the effect of a cognitive intervention [50, 51], and one trial investigated the effect of a dual-task intervention [52] on cognitive function in T2DM.

### Characteristics of studies

The six included exercise trials were published between 2010 and 2018 (Table 1). In total, 604 participants (43.7% males) were allocated to either an exercise intervention or concurrent control. Study sample sizes ranged between 16 and 415. All trials [44–49] recruited patients with T2DM. Three trials [45–47] used a multimodal exercise design incorporating aerobic exercise, resistance/strength training, flexibility and balance training. The remaining three trials [44, 48, 49] used aerobic training only. The duration of exercise interventions ranged from 2 to 6 months with the exception of one trial [46] which lasted 24 months. The frequency of exercise training ranged from 2 to 8 sessions per week, with the duration of training sessions lasting between 30 minutes and 60 minutes. Further details of all exercise trials are given in Table 1.

**Table 1:** Characteristics of included exercise, cognitive, and dual-task trials.

Study (country)	Population	Intervention	Comparison	Cognitive outcomes
Exercise trials				
Baker et al. (2010) (USA) [44]	IGT and T2DM	6 months	Stretching and balance exercise.	Trail making test
	Total *n =* 28	Aerobic exercise		Task switching
	Int: *n* = 19	4 x/wk		Stroop colour-word interference
	Con: *n* = 9	45–60 mins		
	Males: *n* = 10	75–85% HR reserve		SOPT
	Age: 71 ± 7.5 (Int)			Verbal fluency
	66 ± 6.0 (Con)			Story recall
				List learning
Callisaya et al. (2017) (Australia) [45]	T2DM	6 month	Stretching and gentle movement.	Global composite scores
	*n =* 50	Multimodal exercise		Victoria Stroop test
	Int: *n =* 26	2 x/wk		Trail making test
	Con: *n =* 24	60 mins		DSC
	Males: *n =* 38	Resistance 14–17 RPE		Digit span (WAIS-III)
	Age: 65.3 ± 5.0 (Int) 67.1 ± 4.8 (Con)	Aerobic 12–13–14–16 RPE		HVLT-R
				Rey Complex Figure Copy and Delay
Espeland et al. (2017) (USA) [46]	T2DM	24 months	Education workshops, stretching, flexibility.	3MSE,
	*n =* 415	Multimodal exercise		DSC (WAIS-III),
	Int: *n* = 199	5–6 x/wk		HVLT-R
	Con: *n =* 216	50 mins		*n*-back task
	Males: *n =* 155	Aerobic 13 RPE		Task switching paradigm
		Resistance 15–16 RPE		Eriksen flanker task
Kour et al. (2015) (India) [47]	T2DM	2 month	Dietary and medication	Stroop test (congruent)
	*n =* 60	Multimodal exercise		Stroop test (Incongruent)
	Int: *n =* 30	Aerobic—5 x/wk		
	Con: *n =* 30	30 mins		
	Males: n = 24	70–80% HRmax		
	Age 35.6 ± 3.72 (Int) 36.4 ± 3.89 (Con)	Resistance—3 x/wk, 3		
		sets, 8–10 reps		
Pisabarro et al. (2018) (Uruguay) [48]	T2DM	5 month	Advised to walk	Adenbrooke cognitive exam (ACE)–Spanish version
	*n = 35*	Aerobic exercise		
	Int: n = 16	6 x/wk		
	Con n = 19	45 minutes		
	Males: n = 26	Moderate/vigorous		
	Age 64.06 ± 5.45 (Int) 62.68 ± 7.09 (Con)	intensity		

Yanagawa et al. (2011) (Japan) [49]	T2DM	3 month programme	Did not specify	MMSE
	*n =* 16	Aerobic exercise		Japanese Stroop test,
	Int: *n =* 9	4 x/wk		Word recall
	Con: *n =* 7	45 mins		digit symbol
	Males: *n =* 11			Trail making test
	Age: 71.56 ± 3.84 (Int) 70.14 ± 3.84 (Con)			Immediate recall
				Delayed recall

Cognitive trials				
Paulo & Yassuda (2012) (Brazil) [50]	Diabetic	Psychoeducational cognitive training 8 training sessions	Did not specify	Verbal fluency
	Total *n =* 35			The short cognitive test
				RBMT
	Int: *n =* 19			
	Con: *n =* 15			
	Males: *n =* 14			
Whitelock et al. (2018) (UK) [51]	T2DM	WM training	Passive control	Working memory capacity
	Total *n =* 81	25 sessions		Attention switching task
	Int: *n =* 45	Completed in 25–50 days.		Paired associates learning
				Spatial span
	Con: *n =* 36	Difficulty closely followed WM capacity		Spatial working memory
	Males: *n =* 47			
	Age: 59.69 ± 8.77 (Int) 62.14 ± 10.29 (Con)			
Dual-task trials				
Shellington et al. (2018) (Canada) [52]	T2DM	6 month	Wait-list	Memory (Monkey ladder, spatial span, digit span, paired associates)
	Total *n =* 25	Square stepping		
		exercise		
	Int: *n =* 12	2 x /wk		Reasoning (Verbal reasoning, double trouble, odd one out)
	Con: *n =* 13	60 mins		
	Male: *n =* 38	Progressive difficulty		
	Age: 65,9 ± 5.2 (Int)			Concentration (Rotations featured match, interlocking polygons)
	71.2 ± 6.9 (Con)			
				Planning (Tree task, token search)
				Antisaccade reaction

T2DM = Type 2 diabetes mellitus, IGT = Impaired glucose tolerance, Int = Intervention group, Con = control group, x/wk = times per week, Rate of perceived exertion scale, mins = Minutes, HRmax = Maximum heart rate, HR reserve = HR reserve, WM = Working memory, DSC = Digit symbol coding, WAIS-III = Wechsler adult intelligence scale–third edition, HVLT-R = Hopkins verbal learning test-revised, 3MSE = Modified mini-mental state examination, SOPT = Self ordered pointing test, RBMT = Rivermead Behavioural Memory Test.

The two included cognitive trials were published in 2012 and 2018 (Table 1). One trial [51] randomised 81 individuals diagnosed with T2DM (60% males) to either a working memory training intervention or a concurrent control. The trial intervention required participants to complete 25 online working memory training sessions within 25 to 50 days. Training tasks included letter span task, backwards digit span task, and a visuospatial task. The training was individualised whereby the difficulty would increase for every two correct answers or decrease for every two incorrect answers. A follow up assessment was included 3 months post intervention. The remaining trial [50] allocated 34 individuals (59% male) to either a psychoeducational training intervention or concurrent control. The intervention consisted of 8 cognitive psychoeducational training sessions. The cognitive training components included tasks based upon auditory memory, visual attention, verbal fluency, memory and ordination. The intervention length, frequency, duration, or difficulty of training was not specified.

The only dual-task trial [52] randomised 25 individuals (68% males) aged ≥50 diagnosed with T2DM to either an intervention or concurrent control (Table 1). The intervention consisted of a 6 month squared stepping exercise involving a visuospatial working memory task cued with a stepping response. Participants were shown a stepping pattern across a gridded mat containing 40 squares in which they were required to memorise and repeat the demonstrated pattern 4 times before moving on to a novel pattern. Sessions were performed twice a week lasting 1 hour in duration. The task difficulty progressed when 80% of participants performed the task correctly. No follow up assessments were included.

### Adherence

Only two out of the six exercise trials [45, 46] reported on exercise adherence. In one study [45] attendance to exercise classes was 79% in the intervention group, whilst the attendance of control participants to the light stretching and gentle movement programme was 75%. Of those who attended exercise classes in the intervention group, only 75% adhered to the full 60 minutes of exercise. In the remaining exercise study [46], the attendance of individuals in the exercise group and health education control were 67% and 81%, respectively. Only one out of the two cognitive trials reported on adherence [51], reporting that only two participants completed <20 out of 25 working memory training sessions in the intervention group and only one participant completed <20 out of 25 passive working memory training sessions. In the one dual-task trial [52] only 4 of the 12 participants in the intervention group attended >50% of the square stepping exercise programme, the remaining 8 attended <40% of sessions. The average attendance of those participants who attended >50% of sessions was 70.2%.

### Measurements of cognition

For the purpose of our analysis, this section will focus on outcomes included in meta-analyses only. Three trials investigated the effects of exercise on sub-domains of executive function. Inhibition response was measured in three trials using the Victoria Stroop task [45], the Japanese version of the Stroop color-word test [49], and the Stroop test [47]. Working memory was measured in two trials using the digit symbol test [49] and digit symbol coding [45]. General executive function was measured in two trials using the trail making test part A and part B [45, 49]. Two trials investigated the effects of exercise on explicit memory, a sub-domain of memory, using paragraph recall [49] and the Hopkins verbal learning [45] test for both immediate and delayed recall. Outcome measures of trials that were included in the review but excluded from meta-analyses can be found in Table 1.

### Risk of bias assessment

The overall risk of bias varied across included trials (Table 2). Only one trial [45] was judged to have had a low overall risk of bias. The remaining eight trials were deemed to have had a moderate or high risk of overall bias.

**Table 2:** Risk of bias assessment of included trials.

Study	Random sequence generation	Allocation concealment	Blinding of outcome assessment	Incomplete outcome data	Selective bias	Overall
Baker et al. [44]	Unclear	Unclear	Low	Unclear	High	**High**
Callisaya et al. [45]	Low	Low	Low	Low	Low	**Low**
Espeland et al. [46]	Low	Unclear	Low	Unclear	High	**High**
Kour et al. [47]	Low	Unclear	Unclear	Low	Low	**Moderate**
Paulo & Yassuda [50]	Unclear	Unclear	Unclear	Unclear	High	**High**
Pisabarro et al. [48]	Low	Low	Unclear	Unclear	Low	**Moderate**
Shellington et al. [52]	Low	Unclear	High	Low	Low	**Moderate**
Whitelock et al. [51]	Low	Unclear	Low	Unclear	Low	**Moderate**
Yanagawa et al. [49]	Unclear	Unclear	Unclear	Low	Low	**High**

### Exercise interventions

**Fig 2:**
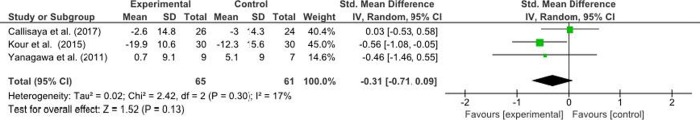
Trial level data, effect estimates and forest plot for the effects of exercise on the Stroop task.

Three trials [45, 47, 49] reported the effect of exercise on the Stroop task. There were 65 individuals in the experimental group and 61 in the control group. The point estimate of effect indicated a greater reduction in the time taken to complete the Stroop task in two of the included trials [47, 49]. Pooled analysis from the three trials demonstrated a small, favourable but not statistically significant effect of exercise on the time taken to complete the Stroop task (SMD = -0.31, 95% CI -0.71–0.09, *P* = 0.13, Fig 2). A low level of statistical heterogeneity was detected among trial level effect (I^2^ = 17%). Compared to other trials included in the meta-analysis, one trial had a high risk of bias [49]. Removal of Yanagawa et al. did not change the overall effect (-0.28, -0.85–0.30, *P* = 0.35).

**Fig 3:**
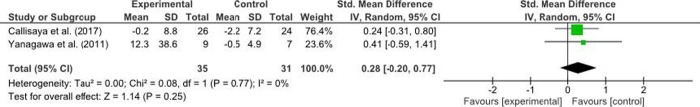
Trial level data, effect estimates and forest plot for the effects of exercise on the trail making test (A).

**Fig 4:**
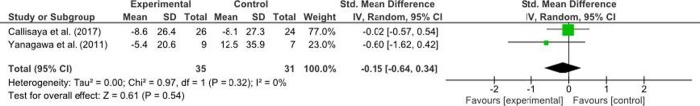
Trial level data, effect estimates and forest plot for the effects of exercise on the trail making test (B).

**Fig 5:**
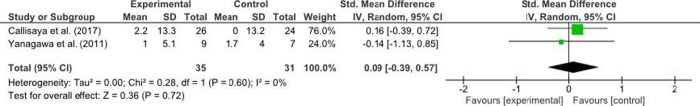
Trial level data, effect estimates and forest plot for the effects of exercise on digit symbol.

**Fig 6:**
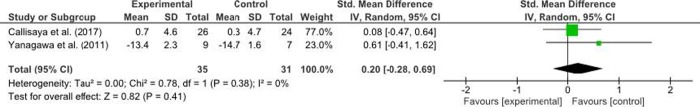
Trial level data, effect estimates and forest plot for the effects of exercise on immediate recall.

**Fig 7:**
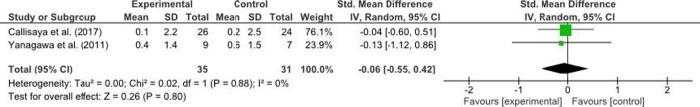
Trial level data, effect estimates and forest plot for the effects of exercise on the delayed recall.

Two trials [45, 49] reported the effect of exercise on the trail making test part A and B, digit symbol, immediate recall, and delayed recall. There were 35 individuals in the experimental group and 31 in the control group. Pooled analysis indicated no significant effects of exercise on the trail making part A (0.28, -0.20–0.77, *P* = 0.25, Fig 3), the trail making part B (-0.15, -0.64–0.34, *P* = 0.54, Fig 4), digit symbol coding (0.09, -0.39–0.57, *P* = 0.72, Fig 5), immediate recall (0.20, -0.28–0.69, *P* = 0.41, Fig 6) or delayed recall (-0.06, -0.55–0.42, *P* = 0.80, Fig 7). There was no evidence of heterogeneity across all measures (I^2^ = 0%). Only two trials [45, 47] provided post intervention outcome data (the Stroop task) for inclusion in a meta-analysis. The Synthesised data from these two studies demonstrated a significant between group effect favouring exercise on the time taken to complete the Stroop task (SMD -0.85, 95% -1.24 – -0.45, *P =* 0.0001). However, a substantial level of statistical heterogeneity was detected (I^2^ = 69%).

Three of the six exercise trials retrieved [44, 46, 48] were not included in the meta-analyses due to the absence of means and standard deviations or group mean differences, and the lack of comparable cognitive outcomes. Individual trials reported statistically significant effects of exercise on cognitive tasks including the trail making test part B (*P* = 0.04), task switching (*P* = 0.03), the Stroop task (*P =* 0.04) [44], digit symbol coding (*P* = 0.05), Hopkins verbal learning test-revised (*P* = 0.005), the Ericksen flanker test (congruent and incongruent *P =* 0.005, *P =* 0.006) [46], and the Adenbrooke cognitive exam Spanish edition (*P* = 0.031) [48].

### Cognitive interventions

Two cognitive trials were retrieved but a meta-analysis could not be conducted due to differences in reported outcomes. The two trials reported statistically significant effects of cognitive training on cognitive tasks including trained working memory capacity (0.99, 0.53–1.46), updating ability (-0.41, -0.85–0.03) [51], the short cognitive test memory score (-0.54, -1.22–0.14) and total score (-0.92, -1.63 – -0.22) [50].

### Dual-task interventions

Only one dual-task was retrieved. The trial reported statistically significant effects of dual-task training on tasks of planning, including the tree task (-0.41, -1.30–0.48) and token search (0.92, -0.01–1.85) between weeks 12 and 24 only. No other cognitive outcomes were statistically significant [52].

## Discussion

The review identified nine trials that met the study inclusion criteria, including one dual-task trial [52], two cognitive trials [50, 51] and six exercise trials [44–49]. The overall quality of included trials was mixed, with the majority of trials having a moderate to high risk of bias. A lack of common outcomes and insufficient number of trials limited the meta-analysis to exercise trials only. Small to moderate effect sizes that favoured the experimental group were identified in tasks of executive function and memory including the Stroop task (-0.31), trail making test part A (-0.28), and immediate recall (0.20), but were not statistically significant. Whilst a meta-analysis could not be conducted using cognitive [50, 51] or dual-task [52] trials, individual trial data were shown to favour these interventions on tasks of global cognition, executive function, and memory.

### Comparison with other reviews

The author is aware of four recent systematic reviews [37–40] that critically appraised the effects of exercise on cognition in T2DM but did not statistically quantify the findings of studies into a single numerical estimate of effect. Two reviews [37, 38] present findings to support the effects of exercise for improving cognitive performance in T2DM, whereas, the two most recent reviews [39, 40] suggest that the strength of the current available evidence does not support these conclusions. The inconsistencies in the findings between previous reviews are most likely attributed to the variations in eligibility criteria, in which the inclusion of trials with differing trial designs is evident. The current review is the first to synthesise quantitative data from interventional trials assessing the effects of exercise training on cognition in T2DM. Whilst we have identified additional studies [45, 47, 48] in comparison to previous reviews, limited availability of data resulted in only a small number of studies being included within the meta-analyses. The present meta-analyses do provide an indication of the effect size of exercise interventions on cognition in T2DM. Small to moderate effects that favoured the experimental group were shown in tasks of executive function and memory including the Stroop task (SMD = -0.31), the trail making test part B (-0.28), and immediate memory (0.20). The observed effect sizes may be practically important in this population [41], but were not significant due to the small sample size of included trials (the Stroop task *n =* 126, trail making part A and Immediate recall *n =* 66). It could be argued that the effect (magnitude) of trials are evident, but the power (precision) to detect them as statistically significant is lacking. Interestingly, individual findings from the exercise trials included in the present meta-analyses indicate that the improvements observed in blood glucose levels, HbA1c, and BMI were significantly associated with improvements in tasks of executive function [47, 49]. In agreement with previous reviews [37], our findings also suggest that the beneficial effects of exercise on cognition may be most significant in domains of executive function, which are possibly mediated through improvements in glucose control and body mass. Previous evidence has shown exercise training to have a beneficial impact on cognition through several mechanisms directly and indirectly related to glucose control. For example, Baker et al. reported improvements in executive function and insulin sensitivity after a 6 month of aerobic exercise training, reflecting the potential benefit of improved glucose metabolism on cognitive processes [44]. Other mechanisms identified through which exercise may improve cognition, indirectly related to improvements in glucose control, include enhanced cerebral perfusion, increased neurogenesis and synaptogenesis, reduced inflammation, increased availability of neurotrophins and neurotransmitters, and reduced cerebral atrophy [28–32].

### Strengths and limitations

The review followed a pre-specified protocol using appropriate methods to identify, examine and synthesise relevant evidence. A rigorous search for published and unpublished trials, involving several electronic databases and scanning of bibliographies, yielded six exercise trials [44–49], two cognitive trials [50, 51], and one dual-task trial [52]. A strength of the review is that it is the first to provide a meta-analysis synthesising the effects of exercise on cognition in T2DM. The review is also the first comprehensive search and evaluation of trials investigating the effect of cognitive and dual-task training on cognition in T2DM. The authors recognise that three exercise trials [44, 46, 48] and two cognitive trials [50, 51] met the pre-specified eligibility criteria but were not included in the meta-analyses. The reason for the exclusion of these trials included a lack in common outcome measures and/or the absence of means and standard deviations or group mean differences. In the case of missing data, all authors were contacted and the retrieval of additional data beyond the published literature is considered a strength of the review.

The lack of common outcome measures was identified as a limitation of the current review that restricted the number of outcomes included in the meta-analyses of exercise trials and precluded the combination of trial findings by meta-analysis in cognitive trials. As addressed in several previous reviews in T2DM [38, 40, 54, 55], there is a need for consensus on cognitive assessments. The use of cognitive tasks that differ in format but measure the same cognitive domain makes it difficult to directly compare the results from different trials. The authors are aware that there are a range of cognitive measures available for a comprehensive assessment of cognition, however, the overwhelming number of neuropsychological tests available (varying in format and complexity) and the lack of guidelines for researchers make comparative analysis of trials difficult. A further limitation were the inconsistencies in the methods used to report data, which precluded the inclusion of trials in the meta-analyses. Baker et al. reported data using Cohen’s F value whereas Espeland et al. reported adjusted data only and so could not be compared with trials that reported data as means and standard deviations or group mean differences [44, 46].

The small sample sizes of included studies in the meta-analyses reduces the precision of findings and widened the confidence intervals for the point estimate of effect. This combined with the limited number of trials included in the meta-analyses reduces the strength of our conclusions with respect to the effect of exercise. In addition, the overall quality of evidence was considered poor. All of the trials included in the review were classified as having an overall moderate to high risk of bias, with the exception of one [45]. The majority of risk of bias domains were graded as unclear, and was primarily a result of poor reporting practice. In addition, the adherence to interventions was also underreported, with only four out of the nine trials [45, 46. 51, 52] included in this review reporting adherence rate. Providing adherence data is important in the context of interpreting trial findings, as it can affect the magnitude of treatment effect and also provide an indication of the acceptability of an intervention [56].

The differences in trial design is also a limiting factor that may have contributed to non-significant findings of the current meta-analyses of exercise trials. The intervention length of some trials was short [47, 49], and may have lacked the sufficient programme length needed to elicit neuropsychological adaptations. A previous meta-analysis [21] evaluated the relationship between exercise and cognition and reported that interventions lasting 6 months or more are most likely to have a greater effect on cognitive performance compared to shorter interventions. In addition, the type of exercise used also differed between exercise trials, with previous research indicating that combined aerobic and resistance training may produce greater benefits on cognition, fasting blood glucose, insulin sensitivity, and body mass compared to aerobic or resistance training alone [21, 22, 57]. The age of participants was also shown to vary between trials, ranging from young adults [47] to older adults [45, 49]. Age is a significant factor that drives cognitive decline with task performance shown to be worse in older adults [58]. It is plausible that the non-significant findings observed in the current study may have been influence by the differences in participant age between trials. Finally, there was also a lack of follow up assessments across included trials. To date, limited evidence exists regarding the continued effects on cognition following the termination of exercise, cognitive, or dual-task training in T2DM.

### Implications for future research

The precision of study estimates in the meta-analyses were considered low, primarily as a result of the small sample size of included trials, causing wider confidence intervals for the point estimate of effect. Only one trial [51] included in the review reported *a priori* power analysis. When exploring the effect of working memory training on measures of cognition, Whitelock et al. conducted a sample size calculation which predicted a sample size of 48 participants in total (24 per group). Future trials should aim to conduct and report an appropriate *a priori* power sample size calculation. Based on a small to moderate effect size (0.3) [59], as shown in the present meta-analyses of exercise trials on the Stroop task and trail making part A, a sample size of 352 (176 in each group) is suggested to detect between group differences in future trials using a power of 0.8 and significance level of 0.05. The duration of exercise trials included in the meta-analyses were relatively short, and discrepancies in the modality of exercise used was evident between trials. Recommendations for the design of future trials include exploration of intervention duration with the incorporation of both aerobic and resistance training performed at a moderate intensity. Future researchers should also look to develop a set of core guidelines to help standardise cognitive outcomes in diabetes research. The lack of homogeneity across cognitive outcomes identified in the current review made it difficult to compare trial findings. Attention should be given to those domains that have been shown to be clinically important in a T2DM population e.g. executive function, visual and verbal memory, attention, processing speed and motor function [10–12]. Furthermore, a notable problem associated with T2DM is the lack of intervention to prevent or slow the progression of cognitive decline, especially in those who present premorbid cognitive deficits but do not differ statistically from those without diabetes. It is therefore important to identify the cognitive domains and neuropsychological tests that are most sensitive to cognitive decline in T2DM, as well as those that are most sensitive to change in response to non-pharmacological interventions such as exercise and cognitive training. In addition, previous evidence has shown that exercise and cognitive training may improve cognition in healthy cohorts through several mechanism including an increase in neurotrophins and neurotransmitters, enhanced cerebral blood flow, reduced inflammation, and through adaptations to the structure of the brain [28–33]. Future studies would also greatly benefit from elucidating the response of biomarkers and neuroimaging correlates of brain health in response to exercise and cognitive training in T2DM. Finally, the overall risk of bias of the evidence was considered moderate to high. This may be a result of poor reporting practices. Future research should be reported using appropriate reporting guidelines (e.g. CONSORT) to increase confidence in the validity of reported results.

## Conclusion

There is a growing evidence base regarding trials investigating the effect of exercise, cognitive and dual-task interventions on cognition in T2DM. Due to a small number of studies retrieved, a meta-analysis was limited to exercise trials only. Synthesised data from exercise trials showed small to moderate effect sizes for improving tasks of executive function and memory, which whilst not significant warrants further investigation into the practical implications of these findings. Despite no meta-analysis, individual cognitive and dual-task trials reported a positive effect of these types of interventions on cognition in T2DM. Further exploration into the effects of exercise, cognitive, and dual-task on cognition is needed in T2DM to help further clarify their effects in this population. Future trials should be developed that include a RCT design that are sufficiently powered to detect small but potentially clinically meaningful differences.

## Supporting information

S1 File PRISMA checklist. (DOC)

S2 File Electronic search strategy. (DOCX)
