# Supplementary material for: Effects of exercise, cognitive, and dual-task interventions on cognition in type 2 diabetes mellitus: A systematic review and meta-analysis
Source: PLoS One. 2020 May 14;15(5):e0232958. doi: 10.1371/journal.pone.0232958 (PMC7224461; doi:10.1371/journal.pone.0232958)
Supplement: S2 File — (DOCX) [file pone.0232958.s002.docx]

PubMed: full electronic search strategy (from inception to March 2020)

1. mh. Type 2 diabetes mellitus
2. Type 2 diabetes
3. Type II diabetes
4. mh. Adult-onset diabetes mellitus
5. Adult-onset diabetes
6. mh. Non-insulin dependent diabetes mellitus
7. Non-insulin dependent diabetes
8. mh. Maturity onset diabetes mellitus
9. Maturity onset diabetes
10. Late onset diabetes
11. mh. Slow onset diabetes mellitus
12. Slow onset diabetes
13. Diabetic
14. T2DM
15. t2d
16. mh. NIDDM
17. 1 OR 2 OR 3 OR 4 OR 5 OR 6 OR 7 OR 8 OR 9 OR 10 OR 11 OR 12 OR 13 OR 14 OR 15 OR 16
18. mh. Exercise
19. Exercise
20. Exercise training
21. Exercise intervention
22. mh. Aerobic exercise
23. Aerobic exercise
24. Aerobic training
25. mh. Physical activity
26. Physical activity
27. mh. Physical exercise
28. Physical exercise
29. Physical training
30. Exergaming
31. Cognitive training
32. Cognitive intervention
33. Cognitive remediation
34. Brain training
35. Computerized training
36. Memory training
37. Attention training
38. Dual task
39. Dual task training
40. Dual task intervention
41. Dual-task
42. Dual-task training
43. Dual-task intervention
44. Motor-cognitive
45. Multi-task
46. Divided attention
47. 18 OR 19 OR 20 OR 21 OR 22 OR 23 OR 24 OR 25 OR 26 OR 27 OR 28 OR 29 OR 30 OR 31 OR 32 OR 33 OR 34 OR 35 OR 36 OR 37 OR 38 OR 39 OR 40 OR 41 OR 42 OR 43 OR 44 OR 45 OR 46
48. mh. Cognition
49. Cognit*
50. Neurocognitive function
51. Brian funct*
52. Mild cognitive impairment
53. Mini-mental state examination
54. Global cognitive function
55. mh. Executive function
56. Executive function
57. Memory
58. mh. Attention
59. Attention
60. 48 OR 49 OR 50 OR 51 OR 52 OR 53 OR 54 OR 55 OR 56 OR 57 OR 58 OR 59
61. 17 AND 47 AND 60
